# The burden and risk factors of Sexually Transmitted Infections and Reproductive Tract Infections among pregnant women in Zimbabwe

**DOI:** 10.1186/1471-2334-10-127

**Published:** 2010-05-21

**Authors:** Nyaradzai E Kurewa, Munyaradzi P Mapingure, Marshal W Munjoma, Mike Z Chirenje, Simbarashe Rusakaniko, Babill Stray-Pedersen

**Affiliations:** 1 Department of Obstetrics and Gynaecology, University of Zimbabwe, Harare, Zimbabwe; 2 Faculty of Medicine, University of Oslo, Oslo, Norway; 3 Department of Community Medicine, University of Zimbabwe, Harare, Zimbabwe; 4 Division of Obstetrics and Gynaecology, Rikshospitalet, University of Oslo, Oslo, Norway

## Abstract

**Background:**

Sexually transmitted infections (STIs) and Reproductive tract infections (RTIs) are responsible for high morbidity among women. We aim to quantify the magnitude of the burden and risk factors of STI/RTI s among pregnant women in Zimbabwe.

**Methods:**

A cross sectional study of pregnant women enrolled at 36 weeks of gestation from the national PMTCT program. Study was conducted from three peri-urban clinics around Harare Zimbabwe offering maternal and child health services.

**Results:**

A total of 691 pregnant women were enrolled. Prevalence of HSV was (51.1%), HIV (25.6%) syphilis (1.2%), *Trichomonas vaginalis *(11.8%), bacterial vaginosis (32.6%) and Candidiasis (39.9%). Seven percent of the women had genital warts, 3% had genital ulcers and 28% had an abnormal vaginal discharge. Prevalence of serological STIs and vaginal infections were 51% and 64% respectively.

Risk factors for a positive serologic STI were increasing age above 30 years, polygamy and multigravid; adjusted OR (95% CI) 2.61(1.49-4.59), 2.16(1.06-4.39), 3.89(1.27-11.98) respectively, partner taking alcohol and number of lifetime sexual partners. For vaginal infections it was age at sexual debut; OR (95% CI) 1.60(1.06-2.42). More than 25% of the women reported previous STI treatment. Fifty two percent reported ever use of condoms and 65% were on oral contraceptives. Mean age gap for sexual partners was 6.3 years older.

**Conclusions:**

There is a high morbidity of STI/RTIs in this cohort. There is need to continuously screen, counsel, treat and monitor trends of STI/RTIs to assess if behaviour changes lead to reduction in infections and their sustainability.

## Background

Sexually transmitted infections (STIs) and Reproductive tract infections (RTIs) are responsible for considerable morbidity in women of fertile age [1].

Zimbabwe is one of the countries in Sub Saharan Africa that is hardest hit by HIV. The epidemic is now declining due to associated behaviour change and a declining prevalence in STIs [2,3]. The high HIV prevalence is associated with histories of STI/RTIs and multiple sexual partners [4-6]. Prevalence of syphilis has been reported to range from 0.5 to 6.7%, HSV from 40 up to 67.1%, Trichomonas vaginalis 9.9 to 21%, whilst gonorrhoea and chlamydia range from 0.9% and 3.9% respectively [5-12].

Pregnant women are the most appropriate target group for studying and identifying factors regarding sexual behaviour and STI/RTIs as they present with evidence of unprotected sex. Sexual behaviours are essential complements to biological evidence in understanding STI/HIV epidemiology. Although there is a link between sexual behaviour and the spread of STI/HIV, this has been reported to be varied and complex [13-15].

It is the changes in behaviours that can be measured which make it possible to elucidate and make conclusions of changes in STI/HIV prevalence. This analysis needs to be updated periodically in order to determine the evidence-based change and inform policy makers appropriately. The aim of the present paper is to quantify the magnitude of the burden of STI/RTIs and risk factors for their acquisition among pregnant women enrolled under a Prevention of Mother to Child Transmission (PMTCT) program in Zimbabwe.

## Methods

A cross sectional study of pregnant women enrolled at 36 weeks of gestation from three peri-urban clinics around Harare Zimbabwe offering maternal and child health services.

These women were recruited from the national PMTCT program where they had been voluntarily counseled and tested for HIV. Trained counselors and midwives verbally invited the women into the study. Midwives explained the study procedure and if the women agreed to participate they then signed an informed consent form.

A study questionnaire was interview administered to source information on socio demographic, lifestyle, sexual and reproductive history, contraception and condom use, current and past medical history and presenting genital infections symptoms.

A doctor or a midwife carried out a physical and gynaecological examination including collection of a high vaginal swab. Venous blood was drawn and for each procedure a verbal explanation was given. Women were included in the study if they were 36 weeks pregnant or more, had come through the national PMTCT program, had been tested for HIV and intended to deliver their index baby at any one of the study sites. They were excluded if they planned to deliver elsewhere or had a history of previous or presenting obstetric complication.

### Laboratory methods

A complete blood count was performed using an automated cell counter. Serum creatinine and liver function tests were carried out. Two HIV-rapid tests, (oraquick and determine) were used to detect HIV seropositivity and in the case of an indeterminant result; a Western blot ELISA analysis was done. If any test was positive, a confirmatory HIV rapid test was done. Syphilis serology was done using plasma reagin assay for screening and Treponema pallidum hemagglutination assay for confirmation. Herpes simplex virus type-2 (HSV-2) antibodies were measured by IgG-based type specific ELISA test (POCkit™ HSV-2) Candidiasis was detected by direct microscopy after addition of 10% KOH. *Trichomonas vaginali*s was demonstrated by wet mount microscopy. Bacterial vaginosis was diagnosed based on Amsells criteria; positive whiff test; pH > 4.5 and detection of clue cells or absence of lactobacilli on wet mount and by Gram stain.

The STI/RTIs were grouped into two main categories:

1) STI positives (based on the serological tests; syphilis, HSV-2 and HIV-1)

2) Vaginal infections comprising of (Trichomoniasis, bacterial vaginosis and candidiasis).

### Treatment for STI/RTIs

Women who presented or were diagnosed as suffering from an STI were provided with treatment and given an appointment to return after one week. They were also given contact slips for their sexual partners encouraging them to visit the study sites for treatment provided by the study.

### Ethical considerations

The local communities, the Medical Research Council of Zimbabwe as well as the Norwegian ethical review committee approved the study.

### Statistical analysis

Data was entered and analyzed using Chicago Illinois Statistical Package for Social Scientists (SPSS) version 12.0. Level of significance was set at p value < 0.05. Unadjusted odds ratios with their 95% confidence intervals were calculated for risk factors of the STIs. Thereafter, risk factors that were significant (p < 0.05) in univariate analysis were entered in multiple logistic regression to calculate adjusted odds ratios.

## Results

A total of 691 pregnant women were recruited and enrolled into the study between April 2002 and October 2003 and responded to the study questionnaire. Of those women that were enrolled, 13(1.9%) did not have a biological sample collected at enrollment and were therefore removed from this analysis.

### Socio-demographic characteristics

Their mean age and standard deviation (s.d) was 24.2 (5.1) years, with the majority 568(82%) having attained at least eight years of primary education, 648(94%) were married and of these 104(10%) were in a polygamous relationship. Only 20% of the women were earning some form of income whereas 30% were frequent travelers. Sixty seven percent of the women had their sexual debut before attaining their 20^th ^birthday. The women reported a mean (s.d) pregnancy rate of 2.1(1.2) and parity 1.0(1.1) respectively.

### Current STI/RTIs

Baseline prevalence of HSV-2 was (51.1%), HIV (25.6%) syphilis (1.2%), Trichomonas vaginalis (11.8%), bacterial vaginosis (32.6%) and candidiasis (39.9%) whilst 81.3. % of the women had a vaginal pH > 4.5. On gynaecological examination, (7%) of the women had genital warts whilst (3%) had genital ulcers and (28%) had an abnormal vaginal discharge. Fifty one percent of the women had a positive serological STI, whilst (64%) had one or more vaginal infections.

Serological STIs increased with age with the highest prevalence being among those above 30 years old OR (95%CI) 3.04(1.78-5.19) compared to those below 20 years of age. Vaginal infections decreased with age, being more prevalent among those below 20 years though not statistically significant.

### History of being treated for STI/RTIs

More than twenty five percent of the women reported having been treated for an STI within the past twelve months. History of having been treated for vaginal discharge was 17.6%, genital ulcers 8.9% and genital warts 7.3%.

### Condom and contraceptive use

More than half of the women (52%) reported ever use of condoms whilst 65% were on oral contraceptives.

### Sexual partners' characteristics

The mean age of sexual partners in this cohort was 6.3 years older than their women counterparts. Thirty three percent of the women alluded to having more than one lifetime sexual partner and less than 5% had more than one partner in the preceding year.

### Risk factors for a positive serological STI

Women above 30 years of age had a higher risk of testing positive for serological STIs whilst they were also associated with being in a polygamous relationship and having an income. Increasing age and polygamy remained strong predictors even after adjusting for other demographic characters. In the multivariate analysis of obstetric factors multigravid was the strong predictor for having a positive serological STI. Adjusted and unadjusted odds ratios and their 95% CI for demographic and obstetric characteristics are demonstrated in Table 1.

**Table 1:** Demographic characteristics as risk factors for serologically tested STIs

Risk factors	Serologic STI Positive	Serologic STI negative	Unadjusted odds ratio (95% CI)	Adjusted odds ratio (95% CI)
*Age in years*	n = 320	n = 302		
<20	50 (40)	74 (60)	1	
20-24	100 (43)	130 (57)	1.14 (0.73-1.77)	1.09 (0.69-1.74)
25-29	96 (61)	62 (39)	2.29 (1.42-3.70)	2.10 (1.28-3.45)
30+	74 (67)	36 (33)	3.04 (1.78-5.20)	2.61 (1.49-4.59)
				
*Type of marriage*	n = 313	n = 285		
Monogamy	277 (50)	272 (50)	1	
Polygamy	36 (73)	13 (27)	2.71 (1.41-5.24)	2.16 (1.06-4.39)
				
*Income generation*	n = 321	n = 299		
No	262 (49)	268 (51)	1	
Yes	59 (66)	31 (34)	1.95 (1.22-3.10)	1.60 (0.95-2.68)
				
*Gravidae*	n = 322	n = 300		
Prima-graviade	75 (34)	145 (66)	1	
Multigravidae	247 (61)	155 (39)	3.08 (2.18-4.34)	3.89 (1.27-11.91)
				
*Parity*	n = 322	n = 300		
No children	86 (36)	151 (64)	1	
More than one child	236 (61)	149 (39)	2.78 (1.96-3.94)	0.61 (0.20-1.91)
				
*History of infant death*	n = 310	n = 298		
No	274 (49)	281 (51)	1	
Yes	36 (72)	14 (28)	2.63 (1.39-4.99)	1.24 (0.54-2.87)
				
*History of stillbirths*	n = 320	n = 298		
No	284 (50)	284 (50)	1	
Yes	36 (72)	14 (28)	2.57 (1.36-4.87)	1.47 (0.64-3.39)
				
*History of contraception*	n = 321	n = 299		
No	87 (38)	142 (55)	1	
Yes	234 (60)	157 (40)	2.43 (1.74-3.40)	0.94 (0.52-1.71)
				
*Ever used condoms*	n = 314	n = 292		
No	161 (48)	174 (52)	1	
Yes	153 (56)	118 (44)	1.40 (1.02-1.93)	1.34 (0.92-1.93)

Serologically tested STIs positive was the outcome in multivariate analysis

Sexual partners of women who tested positive for a serological STI were older compared to those of negative ones, mean age 31.7 and 28.6 years respectively p < 0.001.

Sexual partners' characteristics were significantly associated with a positive serological STI where in the multivariate model it is partner taking alcohol that remained as significant predictor, OR (95%CI) 1.16(1.01-1.33). Women who reported having more than one lifetime sexual partner and having children with different paternity were more likely to test positive for a serological STI even after adjusting for other partner characteristics (Table 2).

**Table 2:** Sexual partners' characteristics as risk factors for serological STIs

Risk factors	Serologic STI Positive	Serologic STI negative	Unadjusted odds ratio (95% CI)	Adjusted odds ratio (95% CI)
*Partner has penile discharge*	n = 301	n = 286		
No	288 (51)	282 (49)	1	
Yes	13 (76)	4 (24)	3.18 (1.03-9.88)	2.74 (0.70-10.75)
				
*Partner has genital ulcers*	n = 301	n = 302		
No	280 (50)	279 (50)	1	
Yes	21 (78)	6 (22)	3.49 (1.39-8.77)	2.22 (0.82-6.0)
				
*Partner takes alcohol*	n = 304	n = 287		
No	122 (44)	156 (56)	1	
Yes	182 (58)	131 (42)	1.78 (1.26-2.49)	1.16 (1.01-1.33)
				
*Violent partner*	n = 310	n = 290		
*No*	188 (48)	200 (52)	1	
*Yes*	122 (58)	90 (42)	1.44 (1.02-2.02)	1.14 (0.78-1.65)
				
*Number of lifetime sexual partners*	n = 319	n = 300		
One only	203 (45)	245 (55)	1	
More than one	116 (68)	55 (32)	2.55 (1.76-3.69)	1.92 (1.21-3.06)
				
*Children have different paternity*	n = 305	n = 281		
No	236 (48)	258 (52)	1	
Yes	69 (75)	23 (25)	3.28 (1.98-5.43)	1.94 (1.04-3.62)
				
*Sexual partners in the past year*	n = 318	n = 300		
One only	304 (51)	297 (49)	1	
More than one	14 (82)	3 (18)	4.55 (1.29-16.02)	1.66 (0.41-6.73)

Serologically tested STIs positive was the outcome in multivariate analysis

### Clinical features for those with a positive serological STI

Women presenting with genital warts and genital ulcers on examination were more likely to test positive for a serological STI, whilst trichomonas vaginalis was the strongest predictor of these infections; OR (95%CI) 3.10(1.71-5.64).

### Risk factors for vaginal infections

Sexual debut before 20^th ^birthday was the most significant predictor for having a vaginal infection even after controlling for other observed and reported symptoms, OR (95% CI) 1.60(1.06-2.42) (Table 3).

**Table 3:** Gynaecologically examined risk factors for vaginal infections

Risk factors	Vaginal Infections Positive	Vaginal Infections Negative	Unadjusted odds ratio (95% CI)	Adjusted odds ratio (95% CI)
*Age debut*	n = 439	n = 237		
>20 years	120 (57)	92 (43)	1	
<20 years	319 (69)	145 (31)	1.69 (1.21-2.36)	1.60 (1.06-2.42)
				
*Vaginal pH*	n = 353	n = 319		
<4.5	56 (52)	51 (48)	1	
>4.5	302 (65)	161 (35)	2.06 (1.35-3.15)	1.67 (1.04-2.68)
				
*Curd-like vaginal discharge*	n = 357	n = 202		
Absent	198 (59)	135 (41)	1	
Present	159 (70)	67 (30)	1.61 (1.13-2.32)	1.54 (1.00-2.38)
				
*Yellow vaginal discharge*	n = 339	n = 208		
Absent	217 (58)	155 (42)		
Present	122 (70)	53 (30)	1.64 (1.12-2.41)	1.71 (1.08-2.72)
				
*Abnormal vaginal discharge*	n = 434	n = 238		
Absent	292 (60)	191 (40)	1	
Present	142 (75)	47 (25)	1.98 (1.35-2.88)	1.59 (0.96-2.60)
				
*Reported genital itchiness*	n = 239	n = 434		
No	251 (59)	174 (41)	1	
Yes	183 (74)	65 (26)	1.95 (1.38-2.75)	1.29 (0.82-2.03)

Vaginal infections positive was the outcome in multivariate analysis

Being in a polygamous relationship was significantly associated with having trichomonas vaginalis; OR (95% CI) 2.24(1.09-4.56). There was no significant association between obstetric characteristics and vaginal infections.

### Clinical features of vaginal infections

Women who presented with clinical warts on examination were more likely to have *bacterial Vaginosis*, OR (95% CI); 2.86(1.45-5.62). Vaginal infections were associated with curd like vaginal discharge and yellow vaginal discharge on examination.

## Discussion

Reproductive tract infections and sexually transmitted infections continue to cause considerable morbidity among pregnant women. Prevalence of these infections in this cohort is high with more than fifty percent of the women presenting with either a positive serogical STI or a vaginal infection. HSV-2 was the most prevalent STI, whilst syphilis was the least prevalent.

HIV prevalence in this cohort is within the range of figures reported in the country at that time [3,18]. Syphilis is a routinely screened infection in pregnancy and if the women had booked for antenatal care in their first trimester there are higher chances that they could have been treated. The prevalence of HSV-2, *T. vaginalis *and BV in our study is consistent with what has been reported in the country, although most of the studies did not report on pregnant populations [5,7,8,15-20]. Prevalence of Candidiasis in this cohort is much higher compared to the 25% reported by Mbizvo in 2001 [5].

High vaginal pH was the strongest predictor of having a vaginal infection. It has been reported that women in Zimbabwe practice vaginal douching, which to a large extend disturb their normal vaginal flora predisposing them to infections [21].

Different predictors were observed for serological STIs and vaginal infections because the former are chronic in nature whilst the latter are acute infections which can be completely cured. Due to the cross sectional design of the study we cannot establish which infection came first, HIV, syphilis or HSV-2 [[8,9,17], and [22]].

Association of increasing age with a positive serological STI are because older women have been sexually active for a longer period, thereby increasing their exposure to infections. Polygamous relationships lead to sexual partner mixing and infidelity, as the women's partners seek other sexual partners or vice versa [19].

Those having an income could have been exposed to infections by offering sexual favours in return for casual work to earn some income. This study was conducted at the time when levels of unemployment were quite high compounded by high inflation in the country [2,23].

High morbidity of vaginal infections among young women and their association with teenage sexual debut is consistent with what has been reported; as their genital tract are not fully developed, this exposes them to higher risk of STI/RTIs acquisition [16,20]. Association of multigravid and multiparity with testing positive for a serological STI is due to longer periods of unprotected sex as couples seek to conceive; this increases their risks of infection.

History of stillbirths and infant deaths' association with STI seropositivity is consistent with studies reporting that maternal STIs have a direct impact on the outcome of the infants [11]. Ulcerative diseases increase risk of contracting HIV and other STIs thus Clinical genital ulcers and warts were positive predictors for testing positive for a serological STI [10,11].

Seventy five percent of reported previous infections predicted testing positive for a serological STI. Self reporting of previous infections is quite high (25%) in this cohort compared to what was reported in another study where none of the women reported any previous infections [11]. Acknowledgement of one's previous infections can be attributed to the positive impact of the counseling that the women receive through PMTCT VCT.

Self-reporting of STI symptoms is a positive observation as it compels one to seek medical treatment early. Seeking prompt treatment for STI symptoms will increase women's chances of being counseled to take an HIV test together with their sexual partners. Once screened for HIV women will then seek to protect themselves from infection and re-infections for those testing positive.

Contraceptive and condom use was quite high in our study, above sixty percent compared to what has been reported both in the Demographic Health Surveys (DHS) and other studies [24].

The association between condom use and testing positive for a serological STI is due to the chronic nature of the infections, where once infected one remains positive throughout their life time. Positive behaviour change after an HIV test has been reported in behavioral studies where those testing positive are likely to use condoms consistently. Use of condoms will in turn protect them against vaginal infections.

Sexual partners' characteristics were strong predictors for a positive serological STI reflecting on the role of sexual partners in fueling these infections. The higher partner age gap is because male partners are sexually active for a longer period with multiple sexual partners which exposes them to infections [[1,5], and [7]]. For a meaningful impact to be achieved in preventing STI/RTI infections, interventions should target health education that involves and promotes positive behavior changes among males.

Women in this study had a considerably high STI/RTI morbidity in pregnancy despite the reported positive behaviour of increased condom use, and reduced number of sexual partners in the preceding year. Two years after PMTCT was initiated, reported positive behaviour change is noted. Our data supports the observed declines in HIV prevalence associated with behaviour changes [3,18].

The strength of this study is that it was conducted at the time when the epidemic had reached a mature stage; characterized with declines in HIV prevalence and incidence attributed to a reduction in STI/RTIs and positive behaviour changes too.

## Conclusion

In this cohort STI/RTIs morbidity is high and that risk factors have remained the same. Positive behaviour is reported in increased condom use and a reduction in number of sexual partners. There is need to continuously screen and monitor trends in STI/RTIs infections and assess whether these behaviour changes are sustained and consistent with reduction in STI/RTIs. Screening and treating women for both serological STIs and vaginal infections are of public health importance and should be reinforced by timeous contact tracing of sexual partners for those infected.

## Competing interests

The authors declare that they have no competing interests.

## Authors' contributions

NEK collected data and drafted the manuscript, MPM participated in data analysis and interpretation of results, MWM carried out the laboratory analysis, CZM supervised data collection, RS supervised data analyses and interpretation of results, BSP designed the study. All authors revised and approved the draft manuscript.

## Pre-publication history

The pre-publication history for this paper can be accessed here:

http://www.biomedcentral.com/1471-2334/10/127/prepub
